# Left main coronary artery vasospasm: A case report of misdiagnosed severe coronary artery disease

**DOI:** 10.1016/j.amsu.2022.103691

**Published:** 2022-04-29

**Authors:** Mahmoud Ismayl, Waiel Abusnina, Noraldeen El yousfi, Ahmed Aboeata, Nattapong Sricharoen

**Affiliations:** a Department of Medicine, Creighton University School of Medicine, Omaha, NE, USA; b Department of Medicine, Division of Cardiology, Creighton University School of Medicine, Omaha, NE, USA; c Department of Medicine, Tripoli Medical Center, Tripoli, Libya

**Keywords:** Coronary artery spasm, Left main coronary artery disease, Coronary CT angiography, Coronary artery bypass grafting surgery, Case report

## Abstract

**Introduction and importance:**

Left main coronary artery (LMCA) vasospasm is rare and can cause demand-supply mismatch that can mimic coronary artery disease (CAD). This could lead to misdiagnosis and inappropriate referral for surgical intervention.

**Case presentation:**

A 55-year-old woman with no cardiac risk factors presented with anginal chest pain. Vital signs were stable and physical exam was unremarkable. Chest x-ray was normal and electrocardiography (ECG) revealed sinus bradycardia with nonspecific ST-segment and T-wave changes in the inferolateral leads present on prior ECGs. Echocardiography revealed a left ventricular ejection fraction of 60–65% without regional wall motion abnormalities and cardiac troponin was within normal limits. Nuclear stress test was unsuccessful due to severe reaction to regadenoson. Subsequent invasive coronary angiography revealed an isolated 70% stenosis of the LMCA. Patient was referred for surgery, however, coronary computed tomography angiography (CCTA) prior to surgery unmasked spasm and prevented unnecessary surgery.

**Clinical discussion:**

Coronary spasm is diagnosed clinically based on typical symptoms, transient ECG changes, and a negative stress test with no regional wall motion abnormalities on echocardiography. During episodes of spasm, coronary angiography would reveal an area of stenosis in the affected coronary segment. This could lead to a misdiagnosis of CAD and, in cases of LMCA stenosis, inappropriate referral for surgical intervention.

**Conclusion:**

LMCA spasm is rare but can mimic CAD leading to misdiagnosis and unnecessary surgery. Physicians should have a high suspicion for spasm especially in patients with anginal chest pain who lack CAD risk factors. CCTA can unmask spasm and prevent unnecessary interventions.

## Highlights

• LMCA spasm is rare but can mimic CAD leading to misdiagnosis and unnecessary CABG.
• Spasm should be considered in patients with angina who lack CAD risk factors.
• CCTA and IVUS can unmask spasm and therefore prevent unnecessary interventions.

## 1 Introduction and Importance

Coronary artery spasm is defined by Wakabayashi et al. as a transient total or subtotal occlusion with electrocardiography (ECG) changes and/or typical chest symptoms [1]. Left main coronary artery (LMCA) vasospasm is extremely rare with a few cases reported in the literature [2]. Catheter-induced LMCA vasospasm during coronary angiography is uncommon but has been reported previously [3]. Based on the coronary artery involved, number of coronaries involved, and the degree and duration of vasospasm, coronary spasm can have a wide range of presentations varying from silent ischemia to unstable angina, myocardial infarction, ventricular arrhythmias, and sudden cardiac death [2]. Our LMCA spasm case is unique in that the patient was initially misdiagnosed with severe left main disease. Based on current ACC/AHA 2021 guidelines, significant left main stenosis carries a class 1 recommendation for coronary artery bypass grafting (CABG) surgery to improve survival, thus our patient was referred for potential surgery [4]. Thankfully, coronary computed tomography angiography (CCTA) unmasked spasm as the underlying diagnosis and prevented unnecessary surgery. Our case aims to build on the current literature supporting the use of CCTA and intravascular ultrasound (IVUS) in cases where coronary artery disease (CAD) is uncertain as they help differentiate between coronary spasm and CAD. It also demonstrates how spasm can mimic CAD angiographically potentially leading to misdiagnosis and unnecessary interventions. The work has been reported in line with the SCARE 2020 Criteria [5].

## 2 Case Presentation

A 55-year-old woman with a past medical history of hypothyroidism presented via ambulance to the emergency department with retrosternal chest pain radiating to the neck and down her left arm that started while she was walking. Pain was associated with nausea and shortness of breath. She denied having similar symptoms in the past. She also denied any orthopnea, paroxysmal nocturnal dyspnea, coughing, lower extremity swelling, palpitation, dizziness, or lightheadedness. The patient didn't have cardiac risk factors including hypertension, diabetes mellitus, dyslipidemia, smoking, or known CAD. She didn't have any surgical history or family history including cardiac disease. She denied the use of alcohol, tobacco, or illicit drugs. Vital signs were stable and physical exam was unremarkable. The differential diagnosis included acute coronary syndrome, pericarditis, perimyocarditis, pneumonia, acute pulmonary embolism, and costochondritis.

**Fig. 1:**
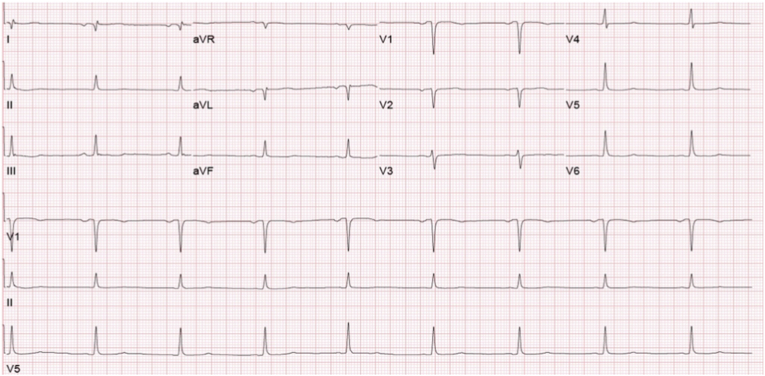
Electrocardiography showing sinus bradycardia with nonspecific ST-segment and T-wave changes in the inferolateral leads.

Chest x-ray was unremarkable and resting ECG revealed sinus bradycardia with nonspecific ST-segment and T-wave changes in the inferolateral leads (Fig. 1) which was present on prior ECGs. Transthoracic echocardiography revealed a left ventricular ejection fraction of 60–65% without regional wall motion abnormalities. Laboratory testing demonstrated normal levels of initial cardiac troponin I and troponin trend for 2 consecutive times 6 hours apart (<0.04 ng/mL; reference range [RR]: <0.04 ng/mL). Thyroid-stimulating hormone level was within normal limits. Lipid profile showed normal levels of cholesterol, high-density lipoprotein, low-density lipoprotein, and triglycerides, and hemoglobin A1C was 5.4% (RR: 4%–5.6%). Complete metabolic panel was unremarkable except for potassium of 3.2 mmol/L (RR: 3.5–5 mmol/L). Complete blood count showed normal cell counts.

**Fig. 2:**
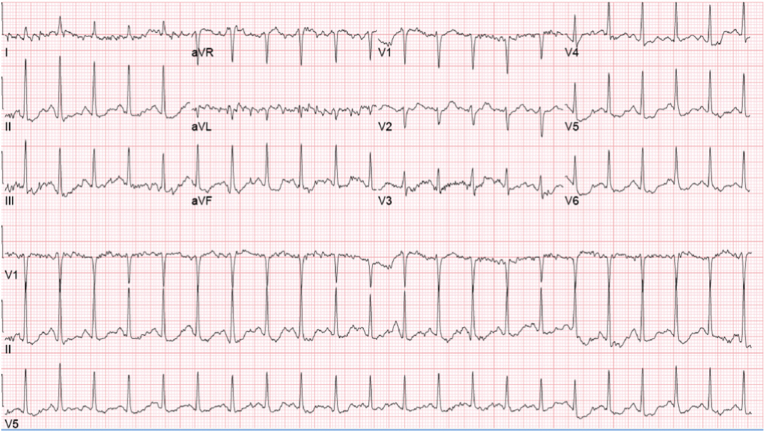
Electrocardiography showing sinus tachycardia with 1 mm ST-segment depressions and T-wave changes in the inferior leads.

Patient was classified as intermediate risk for CAD based on clinical decision pathways and thus underwent a nuclear stress test with regadenoson for ischemic evaluation in the outpatient setting on day 2. Rest images were obtained successfully, however upon administration of the regadenoson, the patient had a severe reaction with shortness of breath, dizziness, and chest pain, which resolved after theophylline administration. ECG after regadenoson injection showed sinus tachycardia with 1 mm ST-segment depressions and T wave changes in the inferior leads (Fig. 2). Patient was admitted to the hospital after an unsuccessful nuclear stress test for further evaluation. Repeat resting ECG showed T-wave inversions in the inferior leads, but no ST changes. Troponin levels on arrival and 2 consecutive times 6 hours apart thereafter were within normal limits (<0.04 ng/mL, RR: <0.04 ng/mL).

**Fig. 3:**
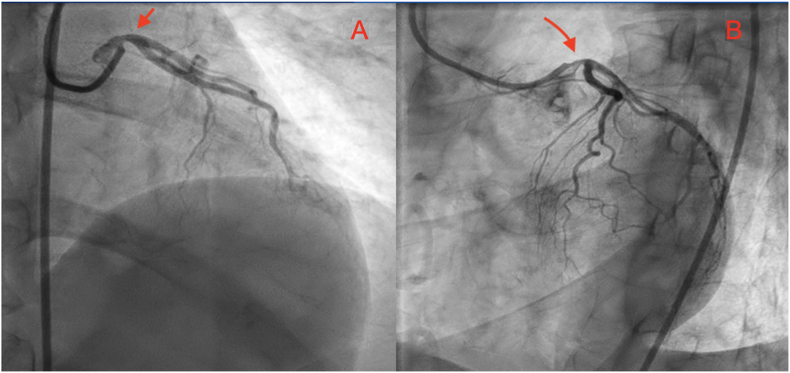
Invasive coronary angiography showing 70% stenosis of the left main coronary artery (red arrow). (A) Right anterior oblique cranial view. (B) Left anterior oblique cranial view. (For interpretation of the references to colour in this figure legend, the reader is referred to the Web version of this article.)

**Fig. 4:**
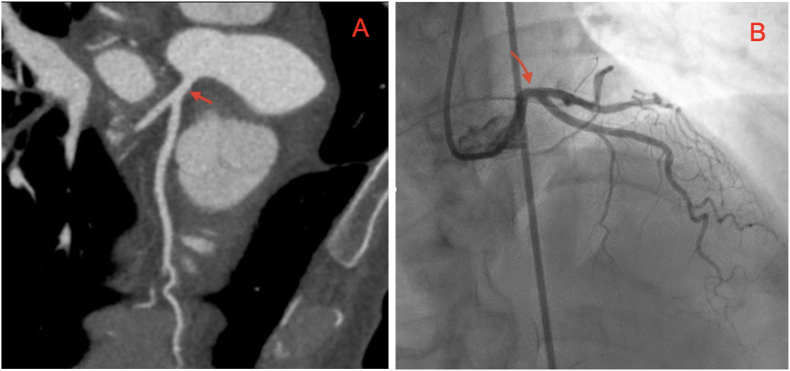
(A) Coronary Computed Tomography Angiography (CCTA) demonstrating the left coronary system with a patent left main coronary artery (red arrow). (B) Invasive Coronary angiography in the right anterior oblique cranial view showing normal left main coronary artery without stenosis (red arrow). (For interpretation of the references to colour in this figure legend, the reader is referred to the Web version of this article.)

Patient underwent invasive coronary angiography on day 3, performed by an interventional cardiology attending, for coronary artery evaluation which revealed 70% stenosis of the distal portion of the LMCA with normal remaining coronaries (Fig. 3). Patient was referred to cardiothoracic surgery for potential CABG surgery on day 4, however given the high suspicion of LMCA spasm in the setting of no CAD risk factors and lack of even mild disease in the other coronaries, the decision was to obtain a CCTA prior to surgery which showed normal LMCA without any stenosis (Fig. 4-A), thus suggesting coronary artery spasm (CAS) as the underlying cause of LMCA stenosis seen during initial coronary angiography. A repeat coronary angiography on day 5 also showed normal LMCA (Fig. 4-B), which further supported the diagnosis of CAS. Patient was diagnosed with LMCA spasm and was managed medically with oral amlodipine 5 mg daily while avoiding unnecessary surgical intervention. She was also advised to avoid known triggers of CAS including smoking. She was adherent with the recommendations and tolerated amlodipine without side effects. The patient was discharged on day 5 with no complications or adverse outcomes and had no more episodes of chest pain at 6-month follow-up in the cardiology clinic.

## 3 Clinical Discussion

CAS is diagnosed clinically based on typical symptoms, transient ECG changes that occur during episodes of chest pain, and a negative stress test with no regional wall motion abnormalities on echocardiography [6]. Many patients who experience recurrent episodes ultimately undergo coronary angiography. However, during episodes of spasm, coronary angiography would reveal an area of stenosis in the affected coronary segment. This could potentially lead to a misdiagnosis of coronary atherothrombotic disease and, in cases of LMCA stenosis such as our case, inappropriate referral for surgical intervention. IVUS during angiography can help identify the lack of significant atherosclerotic disease, which is suggestive of coronary spasm, and therefore prevent misdiagnosis [6]. However, our patient unfortunately did not undergo IVUS. In addition, CCTA may help differentiate between coronary atherosclerosis and coronary spasm [7].

Coronary spasm is treated medically with vasodilators including nitrates and calcium channel blockers [2]. Beta-blocker monotherapy should be avoided as beta-blockade can lead to unopposed alpha-adrenergic stimulation and worsening coronary vasoconstriction [8]. In a previous report on patients with LMCA spasm who underwent CABG surgery, approximately one-third of the left internal mammary grafts got occluded [9]. Therefore, surgical management of coronary spasm is usually unnecessary. Despite this, some patients with coronary spasm who are misdiagnosed with CAD during initial coronary angiography are at risk of undergoing unnecessary CABG surgery. To prevent unnecessary CABG surgery, repeat cardiac catheterization preferably with IVUS is a safe option that helps reevaluate and potentially recognize patients with coronary spasm [10]. However, this approach is invasive and puts the patient at risks related to the procedure. A non-invasive alternative approach is CCTA as it can unmask LMCA vasospasm, as shown in previous studies, and therefore prevent unnecessary CABG surgery [11]. Our patient was initially falsely diagnosed with atherothrombotic LMCA disease after coronary angiography and was referred for possible CABG surgery, however CCTA unmasked the true diagnosis of coronary spasm and prevented the patient from undergoing unnecessary surgical intervention.

## 4 Conclusions

LMCA spasm is rare but can mimic CAD leading to misdiagnosis and potentially unnecessary surgery. Physicians should have a high suspicion for spasm especially in patients with anginal chest pain who lack CAD risk factors. CCTA and IVUS should be considered as they can unmask spasm and therefore prevent unnecessary interventions.

## Provenance and peer review

Not commissioned, externally peer-reviewed.

## Research registration

None.

## Ethical approval

Given the nature of the article, a case report, no ethical approval was required.

## Sources of funding for your research

This research did not receive any specific grant from funding agencies in the public, commercial, or not-for-profit sectors.

## Author contributions

All authors contributed to this manuscript.

Mahmoud Ismayl: Writing - original draft.

Waiel Abusnina: Writing and editing.

Noraldeen El yousfi Rass: Writing - original draft.

Ahmed Aboeata: Supervision; reviewing and editing.

Nattapong Sricharoen: Supervision; reviewing and editing.

## Registration of research studies

This is not an original research project involving human participants in an interventional or an observational study but a case report. This registration was not required.

## Consent

Written informed consent was obtained from the patient for publication of this case report and accompanying images. A copy of the written consent is available for review by the Editor-in-Chief of this journal on request.

## Guarantor

Mahmoud Ismayl, MD.

Mahmoudismayl1995@hotmail.com.

## Declaration of competing interest

The authors have no conflict of interest to declare.
